# The influence of temperature on suicidality in youth: The role of insomnia

**DOI:** 10.1016/j.joclim.2026.100735

**Published:** 2026-09-21

**Authors:** Joshua R. Wortzel, Katherine A. Brown, Francis P. Donnelly, Mary Kathryn Cancilliere, Elizabeth Chen, Kathleen Donise, Nicole R. Nugent

**Affiliations:** a Department of Psychiatry, Institute of Living, Hartford Healthcare, United States; b Department of Psychiatry and Human Behavior, Brown University, United States; c Rhode Island Hospital/Hasbro Children’s Hospital, Providence, RI, 02903, United States; d Emma Pendleton Bradley Hospital, East Providence, RI, 02915, United States; e Center for Biomedical Informatics and Center for Computational Molecular Biology, Brown University, United States; f GIS and Data Services, Brown University Library, United States

**Keywords:** Insomnia, Suicidality, Temperature, Youth

## Abstract

**Introduction:**

Suicide is the second leading cause of death among youth, with growing evidence linking higher ambient temperatures to increased suicidality – a concerning trend as global temperatures rise. Sleep disruption is a theorized mechanism, though clinical evidence is lacking.

**Materials and methods:**

We performed a cross-sectional analysis of 5,590 pediatric patients (3–18 years) presenting to a psychiatric emergency department with and without suicidality (December 2018-March 2023). Seven-day averages of mean and minimum daily ambient temperatures and precipitation were obtained at home addresses from the PRISM Climate Group dataset. Logistic regression assessed insomnia as a moderator and mediator of the temperature-suicidality relationship, controlling for sociodemographic, diagnostic, and environmental variables. Negative controls included modeling suicidality using hypersomnia-temperature and insomnia-precipitation interactions.

**Results:**

Mean and minimum daily temperatures moderated the temperature-suicidality relationship only in those with insomnia (Mean Temperature: RR: 1.06 per 5°C [95% CI 1.01–1.06], p = 0.015; Minimum Temperature: RR: 1.06 per 5°C [95% CI 1.01–1.06], p = 0.010). Minimum temperature, a proxy for nighttime temperature, was associated with suicidality through insomnia in a mediation model (Indirect Pathway: OR: 1.05 [95% CI 1.00–1.11], p = 0.045), though mean temperature was not. No significant moderation occurred modeling suicidality with hypersomnia-temperature or insomnia-precipitation interactions.

**Conclusion:**

This is the first clinical evidence that insomnia influences the heat-suicidality relationship in youth. Limitations include cross-sectional data that cannot establish temporal directionality of associations, heterogeneous suicidality outcomes, self-reported sleep data, and pandemic-era data collection. Larger mechanistic analyses of temperature, sleep, and suicidality are needed to understand climatic mental health impacts and develop interventions.

## 1 Introduction

Warmer temperatures during heat waves and warmer seasons are associated with worse affective symptoms, violence, and suicidality [1,2]. For example, one study of 3.5 million emergency department (ED) visits found that days of extreme heat (95th percentile of county-specific warm-season temperatures) were associated with an 8% increase in ED visits for any mental health condition [3]. Suicidality appears particularly affected. Multinational analyses of over 1.3 million suicides across 12 countries show that higher ambient temperatures are associated with increased suicide risk, with an overall 33% increase during peak temperatures compared to non-peak temperatures [4]. Estimates of this relationship are predicted to increase by 8.3–11.4% by 2050 and 8.5–21.7% by 2090 depending upon how climates continue to change [5].

The heat-suicidality relationship is particularly concerning among youth, a population uniquely vulnerable to heat stress and its neurobiological consequences as well as compounding biosocial burden as climates change. Physiologically, children sweat less efficiently during heat stress and have a higher volume-to-surface area ratio than adults, limiting thermoregulatory capacity during heat stress [6]. Socially, many youth do not have meaningful control over their environments and are more often situated in settings that increase heat risk, including schools with limited climate control, organized outdoor sports programs, and workplaces with fewer heat protections [7]. Those born in 2020 are also estimated to accumulate at least double the lifetime heatwave exposure of those born in 1960 [8], making today's youth a generation with both heightened vulnerability and heightened exposure. Given that suicide ranks as the second leading cause of death in this age group [9], understanding how temperature impacts youth mental health appears particularly important.

Multiple epidemiological studies support the theoretical mental health risks that youth face in relation to ambient heat. In a study of suicide rates among 15–25-year-olds in Istanbul, suicide attempts occurred more frequently during summer months and were positively correlated with the temperature on both the day of attempts and the mean temperature over the 10 days preceding attempts [10]. An analysis of 25 years of US suicide data in 5-24-year-olds found that summer suicide rates rose by 2.68% per degree Celsius increase in average monthly temperatures [11]. Research analyzing emergency department data among 6–18-year-olds in California (2005–2013) and 12–24-year-olds in Australia (2012–2019) independently found that suicidality presentations rose by 1.3% for each 1°C increase in average daily temperature – the highest in any age group – with the strongest associations occurring during warm months [12,13].

Despite this established association, the mechanisms linking heat to suicidality remain poorly understood. The effect of temperature on sleep is a theoretical link [14]. Sleep-wake disturbances are both a risk factor for and a symptom of mood disorders [15], affecting cognitive functions that influence mental health risk. Observational studies show increased ambient heat (particularly above 24–27°C) reduces total sleep time, impairs sleep quality, and leads to increased wakefulness during the biological (circadian) night [[16], [17], [18]]. Data suggest insomnia increases the risk of suicide 3.5-fold in the general population and 18.3-fold in patients with mental illness [19]. This may be because nocturnal wakefulness represents a critical vulnerability period for mental health, as the brain operates at its cognitive and emotional nadirs during these hours, with severe impairments in executive function/hypofrontality (i.e., decreased activity/functional connectivity in the frontal cortex) [20,21], mood regulation [22], and impulse control [23,24], all risk factors for suicidality [25]. While hypersomnia (i.e., excessive sleepiness and prolonged sleep duration) has also been positively correlated with suicidality [26], the relationship between hypersomnia and elevated ambient temperatures has not been established in the literature.

These findings suggest insomnia may operate in two distinct but related ways within the heat-suicidality pathway. As a moderator, insomnia may mark a physiologically and neurobiologically vulnerable group in whom heat-related disruption to executive function and impulse control translates into elevated suicide risk, such that nighttime heat increases suicidality specifically among youth awake to experience it. As a mediator, nighttime heat may cause or worsen insomnia, which in turn produces the neurobiological dysregulation through which suicide risk is elevated, representing a physiological pathway connecting ambient temperature to acute psychiatric crisis. We aimed to conduct an analysis of the interaction of ambient temperature and sleep on suicidality in an acute clinical sample of children and adolescents presenting to an ED in mental health extremis. We further hypothesized that insomnia would moderate and/or mediate this relationship, providing evidence for an insomnia pathway through which nighttime heat influences suicidal crises. As negative controls, we tested whether hypersomnia moderated the temperature-suicidality relationship and whether insomnia moderated a precipitation-suicidality relationship.

## 2 Materials and methods

### 2.1 Clinical data

This cross-sectional study reviewed electronic health records (EHR) augmented by chart review of pediatric patients (3 to18 years old) evaluated at a tertiary academic medical center by a psychiatry emergency service (PES) from December 31, 2018, to March 23, 2023. The data were accessed on March 23, 2023, for research purposes. The highest acuity children are referred to PES for evaluation of emotional and/or behavioral concerns (e.g., severe depression and/or anxiety, aggression/irritability, non-suicidal self-injurious behavior, suicidal ideation, or homicidal ideation). The study dataset included both structured and unstructured EHR data for initial evaluations from pediatric patients who were evaluated by PES [27,28]. These data were obtained in comma-separated value (CSV) format using a custom report through the Epic Reporting Workbench. Of note, this dataset does not include a small subset of patients who were so significantly injured by attempted suicide that they were admitted directly to medical floors without a psychiatric evaluation in the ED. During this period, PES completed 9,623 assessments of 5,590 individuals. Only initial encounters during the assessed time period were used for patients with multiple presentations so that everyone was represented once in the dataset. This study was reviewed and approved by the Institutional Review Board. STROBE guidelines were followed. Given that this was a retrospective study of medical records, the IRB waived the requirement for informed consent, and the data were fully blinded at the beginning of the analysis.

The dataset contained structured data such as demographics, including patient age, legal sex, race, insurance type (private or public, as a proxy of socioeconomic status) [28], home address, date of hospital presentation, and primary problem/diagnosis for insurance. Presentations were assigned an astronomical season based on the date of initial presentation (i.e., Winter: December 21–March 20; Spring: March 21–June 20; Summer: June 21–September 20; Autumn: September 21–December 20). The dichotomous variables suicidality, insomnia, and hypersomnia were determined based on billing diagnosis and initial evaluation notes using rule-based natural language processing techniques [28]. Specifically, information was extracted from clinical notes documenting initial evaluations performed by qualified PES clinicians under the supervision of child and adolescent psychiatrists, following hospital clinical standards. “Suicidality” as an umbrella term was determined using billing diagnoses and note content, including positive suicidal ideation in the mental status exam, explicit documentation of 'suicidal ideation' or 'suicide attempt,' or clinician assessment of 'moderate' or 'high' suicide risk or 'active suicidal ideation,' which are standardized dropdown fields in the note template used by all clinicians in this medical service [28]. Sleep disturbances (insomnia and hypersomnia) were assessed using standardized dropdown fields in the initial evaluation note template asking explicitly about sleep and were coded as dichotomous variables.

### 2.2 Ambient heat and precipitation data

Daily climate data for the United States were obtained from the PRISM Climate Group at Oregon State University [29]. Data are published as GIS rasters at 4 km resolution and include mean temperature in degrees Celsius and total precipitation in millimeters (as a proxy for cloud cover/light exposure [30]. Data are derived from point-based weather stations and interpolated across regions accounting for elevation, slope, proximity to coastlines, and other factors [29]. Python scripts were written to process climate data and relate it to patients’ home longitude and latitude coordinates over the week preceding hospital presentation [31].

We considered multiple methods to approximate temperature exposure. Daily mean temperature has commonly been used to approximate overall thermal exposure, though studies vary greatly [32]. For example, minimum daily temperature has been used to better approximate average evening heat exposure [33,34]. Therefore, we tested both mean and minimum daily temperatures. The duration of temperature exposure also has varied by study, with many assessing same-day temperature to capture acute effects [4], while others have looked at weekly [35] and even monthly [30] temperature averages to assess cumulative and lag effects. We opted to use 7-day average mean and minimum temperatures preceding hospital presentation. Same-day temperatures would have failed to capture heat exposures during the evening before presentation when insomnia would have occurred, and self-reported insomnia likely does not relate to one night of disrupted sleep but rather successive nights of sleep disruption. There is also evidence that higher temperatures produce lagged adverse sleep effects that accumulate over several subsequent days [33]. For ease of nomenclature, 7-day average daily mean temperature will be referred to as ‘mean temperature’ and 7-day average daily minimum temperature will be referred to as ‘minimum temperature’ throughout the remaining manuscript.

### 2.3 Analysis

We conducted logistic regressions with suicidality as the dependent variable and insomnia and weekly average ambient temperature (mean or minimum) both individually and in interaction as independent variables. A likelihood ratio test was used to confirm the significance of the interaction term. Relevant covariates were sequentially added, including potential confounding sociodemographic variables (legal sex, minority, age, and primary insurer as a proxy of socioeconomic status as previously used in this dataset and others) [36,37], the most prevalent mental health diagnoses (depression, anxiety, and Post-Traumatic Stress Disorder [PTSD]), and environmental variables (weekly average precipitation and season). Adjustment for season was considered particularly important given that acclimatization, the improved ability to thermoregulate with sustained exposures to higher temperatures, could differentially influence the effects of temperature in the spring vs the summer or fall [38]. As negative controls, we conducted two parallel analyses of suicidality: one testing the independent effects and interaction of insomnia and weekly average precipitation, and another testing the independent effects and interaction of hypersomnia and weekly average mean ambient temperature. Both analyses included all aforementioned covariates as independent variables.

To further uncover moderator effects in a secondary analysis, we stratified the dataset by cohort with and without insomnia and conducted logistic regression using temperature (mean or minimum) and all sociodemographic, mental health, and environmental covariates described above as independent variables. We then performed mediation analysis with temperature (mean or minimum) as the exposure, insomnia as the mediator, and suicidality as the outcome. To address the possibility of reverse directionality inherent in cross-sectional mediation analysis, a complementary analysis was conducted in which suicidality was modeled as a predictor of insomnia, adjusting for temperature (weekly mean and minimum) and all sociodemographic, diagnostic, and environmental covariates described above. The prevalence of suicidality was graphed by insomnia status and 5°C temperature bins for visualization of the relationship across the temperature spectrum, and marginal effects plots were generated in those with and without insomnia to visualize model fit. All analyses used 5°C temperature intervals to show clinically meaningful effects. Odds ratios (ORs) were calculated by exponentiating the beta coefficients multiplied by the temperature increment. ORs were converted to risk ratios (RRs) using the Zhang-Yu approximation formula [39], RR = OR / [(1 - P₀) + (P₀ × OR)], where P₀ represents the baseline prevalence of suicidality in each stratified group (With Insomnia: 69.2%, Without Insomnia: 50.8%). An α=0.05 was used. The primary outcomes (i.e., two moderation analyses using mean and minimum daily temperature in interaction with insomnia) were Bonferroni corrected (p-values multiplied by 2). All analyses were performed using R Studio statistical programming, version 4.2.0 [40].

## 3 Results

The sample (n = 5,590) included a slightly higher proportion of high-school-age adolescents to school-age and middle-school-age children (54.1%vs 45.9%), birth-female to birth-male individuals (58.4%vs 41.5%), and private to public insured patients (51.9%vs 46.4%) (Table 1). Two-thirds of the sample identified as white compared to non-white (63.1%vs 36.9%). Half of the sample presented with suicidality (55.0%), one in five reported insomnia (22.4%), and one in 14 reported hypersomnia (7.1%). The majority of patients had depression or anxiety (72.8% and 62.5%, respectively), followed by PTSD (17.4%). The number of presentations differed by season, with the highest proportion in winter and the lowest in summer (32.2%vs 17.0%); however, there were 5 winters and only 4 springs, summers, and falls included in the dataset.

**Table 1:** Demographics of sample.

Patient Characteristics	No. (%) (N = 5590)
**Age**	
<14-years-old	2565 (45.9)
14–18-years-old	3025 (54.1)
**Legal sex**	
Male	2321(41.5)
Female	3263 (58.4)
Unspecified	6 (0.1)
**Primary Payer**	
Public	2592 (46.4)
Private	2903 (51.9)
Unspecified	95 (1.7)
**Minority Status**	
White	3530 (63.1)
Non-White	2060 (36.9)
**Sleep**	
Insomnia	1252 (22.4)
Hypersomnia	396 (7.1)
**Suicidality**	
Suicidal Ideation/Plan/Intent	3072 (55.0)
**Mental Health Diagnoses**	
Depression	4070 (72.8)
Anxiety	3491 (62.5)
Post-Traumatic Stress Disorder	975 (17.4)
**Seasons**	
Winter	1800 (32.2)
Spring	1399 (25.0)
Summer	948 (17.0)
Autumn	1443 (25.8)
**Climate Factors**	**Mean (SD)**
Average Weekly Temperature (°C)	9.8 (8.6)
Average Weekly Precipitation (mm)	3.5 (3.4)

**Legend:** SD = Standard Deviation.

In the fully adjusted model (Model 4) including all sociodemographic, diagnostic, and environmental covariates (see Supplemental Table 1 for intermediate models), we observed a significant interaction between ambient temperature (both mean and minimum) and insomnia in predicting suicidality (Mean Temperature: Risk Ratio [RR]: 1.04 per 5°C increase, 95% confidence interval [CI]: 1.01–1.06; p = 0.016; Minimum Temperature: RR 1.04, 95% CI 1.01–1.06, p = 0.014) (Table 2). Interactions between insomnia and precipitation and hypersomnia and temperature were tested as negative controls, which were not significant (Supplemental Table 2).

**Table 2:** The ineraction of ambient temperature and insomnia in modeling suicidality.

Full Model (Model 4)	Full Models (per 5 °C change)				
	Estimate	OR (RR)	RR 95%CI	Z score	p-value
7-day Average Daily Mean Temp * Insomnia	0.12	1.13 (1.04)	1.01–1.06	2.67	0.016
7-day Average Daily Minimum Temp * Insomnia	0.12	1.13 (1.04)	1.01–1.06	2.70	0.014

**Legend:** Row 1 uses suicidality as the dependent variable and insomnia and 7-day average daily mean temperature both separately and in interaction, in addition to sociodemographic, diagnostic, and environmental covariates (Full Model 4 of Supplemental Table 1). The second row uses suicidality as the dependent variable and tests insomnia and 7-day average daily minimum temperature both separately and in interaction in addition to the same covariates. P-values are Bonferroni corrected. OR indicates the odds ratio, RR the risk ratio, and 95% CI the 95% confidence intervals. The reference group for Model 4 was white males <14 years old without insomnia with unspecified insurance in winter.

Stratified analysis of those with (n = 1,252) and without (n = 4,338) insomnia using mean or minimum temperatures and all other covariates to model suicidality further supported moderation (Table 3). Higher temperature was associated with increased suicidality in only those with insomnia (Mean Temperature: RR 1.06 per 5°C increase, p = 0.015; Minimum Temperature: RR1.06, p = 0.010). In those without insomnia, higher temperatures were marginally associated with lower suicidality.

**Table 3:** The relationship between ambient temperature and suicidality (dependent variable) stratified by insomnia status.

A. 7-Day Average Daily Mean Temperature					
Insomnia Status Stratification	Full Models (per 5 °C change)				
	Estimate	OR (RR)	RR 95% CI	Z score	p-value
Without Insomnia	−0.081	0.92 (0.96)	0.92–1.00	−2.05	0.041
With Insomnia	0.20	1.22 (1.06)	1.01–1.10	2.42	0.015

B. 7-Day Average Daily Minimum Temperature					
Insomnia Status Stratification	Full Models (per 5 °C change)				
	Estimate	OR (RR)	RR 95% CI	Z score	p-value
Without Insomnia	−0.072	0.93 (0.96)	0.93–1.00	−1.84	0.066
With Insomnia	0.21	1.23 (1.06)	1.02–1.10	2.59	0.010

**Legend:** Table A uses 7-day average daily mean temperature and Table B uses 7-day average daily minimum temperature. OR indicates the odds ratio, RR the risk ratio, and 95% CI the 95% confidence interval. The reference group for Model 4 was white males <14 years old without insomnia with unspecified insurance in winter.

Minimum temperature mediated the temperature-suicidality relationship through insomnia (OR: 1.05 [95% CI 1.00–1.11], p = 0.045), while mean temperature did not (OR: 1.05 [95% CI 0.99–1.10], p = 0.085) (Table 4). Our complementary reverse directionality analysis modeling suicidality as a predictor of insomnia and adjusting for temperature and all covariates yielded significant associations in both the mean temperature-adjusted (OR: 1.99 [95% CI 1.72–2.31], p < 0.001) and minimum temperature-adjusted (OR: 1.99 [95% CI 1.72–2.31], p < 0.001) models, consistent with the cross-sectional nature of the data and the inability to establish temporal precedence from these analyses (Supplementary Table 3). The percentage of patients presenting to the ED with suicidality was higher above −5°C among those with insomnia than without, with a sharp rise in suicidality rates among those with insomnia above 20°C that was not observed in those without (Fig. 1A). Marginal effects plots support an association between temperature and suicidality rates in those with insomnia and not in those without (Fig. 1B).

**Table 4:** Insomnia as a mediator of the temperature-suicidality relationship.


A. 7-Day Average Daily Mean Temperature per 5 °C					
Models	Per 5 °C increase				
	Estimate	OR	OR 95% CI	Z score	p-value
Path A: Temp -> Insomnia	0.067	1.07	0.99–1.15	1.77	0.077
Path B: Insomnia -> SI	0.689	1.99	1.72–2.31	9.10	<0.001
Path C: Direct Effect	−0.029	0.97	0.91–1.04	−0.81	0.42
Indirect Effect (C') (A*B)	0.046	1.05	0.99–1.10	1.74	0.082

B. 7-Day Average Daily Minimum Temperature per 5 °C					
Models	Per 5 °C increase				
	Estimate	OR	OR 95% CI	Z score	p-value
Path A: Temp -> Insomnia	0.077	1.08	1.00–1.16	2.05	0.040
Path B: Insomnia -> SI	0.689	1.99	1.72–2.31	9.09	<0.001
Path C: Direct Effect	−0.019	0.98	0.92–1.05	−0.55	0.58
Indirect Effect (C') (A*B)	0.053	1.05	1.00–1.11	2.00	0.045

**Legend:** (A) This mediation uses 7-day average mean temperature. (B) This mediation uses 7-day average minimum temperature. In both models, temperature (Temp) is the exposure, insomnia status is the mediator, and outcome is suicidality status. *Pathway a* represents the effect of temperature on insomnia status, *pathway b* the effect of insomnia status on suicidality, and *pathway c’* is the indirect effect of temperature on suicidality through *pathways a and b*, and *pathway c* is the direct effect of temperature on suicidality after accounting for this indirect pathway. All regressions use Model 4 as outlined in Table 2. OR is the odds ratio and 95% CI is the 95% confidence interval.

**Fig. 1:**
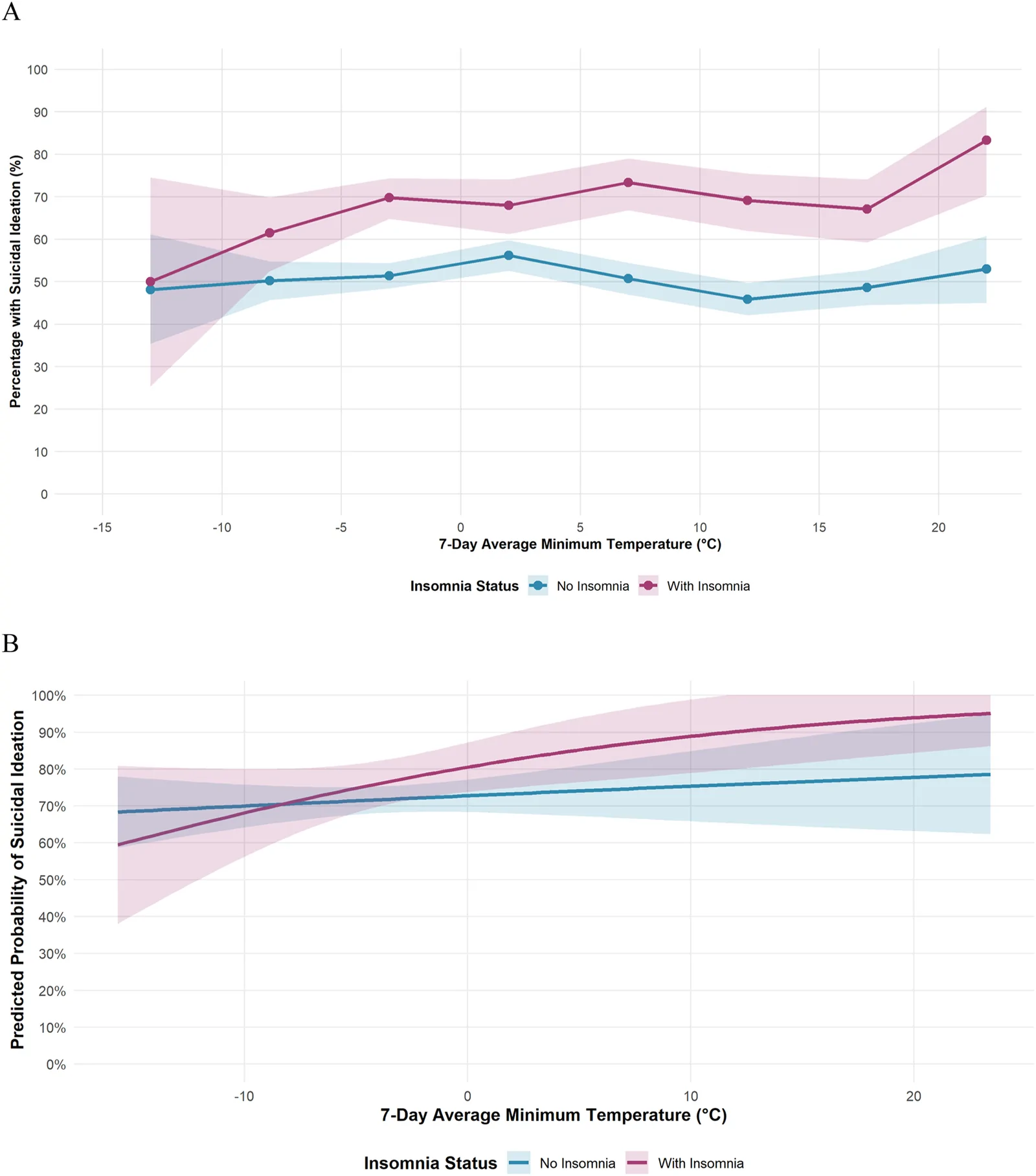
Suicidality prevalence and marginal effects by insomnia status across ambient temperatures.

**Legend: (**A) Suicidality prevalence by temperature and insomnia status. 7-day average daily minimum temperature was binned by 5 °C (N = 4338 for ‘No Insomnia;’ N = 1252 for ‘With Insomnia’). Points show observed percentages in each temperature bin. The shaded areas show 95% confidence intervals. (B) Marginal effects – predicted probability of suicidality by insomnia status. Probability of suicidality was predicted using 7-day average daily minimum temperature and was modeled separately in those reporting insomnia and no insomnia. Each model controls for age, sex, minority status, insurance, mental health diagnoses, season, and precipitation. Shaded areas represent 95% confidence intervals.

## 4 Discussion

This study extends prior epidemiological evidence linking ambient heat to suicidality in youth [[10], [12], [13]] and provides the first clinical evidence that insomnia influences the heat-suicidality relationship in youth presenting to emergency psychiatric care, consistent with prior work that higher ambient temperatures are associated with sleep disruption [17,18] and that insomnia is a potent independent risk factor for suicide [19]. Insomnia moderated the temperature-suicidality relationship, meaning temperature was associated with suicidality only among youth with insomnia. Mediation analysis revealed that minimum temperature, but not mean temperature, was associated with suicidality through insomnia, though the marginal significance of this finding (p = 0.045) and the cross-sectional study design preclude conclusions about complete mediation or it being the definitive causal mechanism. These data are consistent with the hypothesis that warmer nighttime temperatures may influence youth suicidality through sleep disruption.

The difference in the significance of mediation using mean and minimum temperature is intriguing, as minimum temperature serves as a better proxy for nighttime temperature, which is likely more directly relevant to sleep physiology. Graphical depiction of suicidality rates in those with and without insomnia shows the greatest difference between these groups on the hottest weeks. A plausible interpretation is that elevated temperatures worsen insomnia, which subsequently increases suicidality. Experimentally increasing nighttime ambient temperature has been shown to prevent core body temperature suppression, which normally occurs at sleep onset, increases nighttime wakefulness, and decreases the duration of Stage 3, rapid eye movement, and slow wave sleep [41]. As described above, this nocturnal wakefulness represents a period of heightened neurobiological vulnerability, with impairments in prefrontal regulation, mood dysregulation, and diminished impulse control, which may be particularly consequential in youth whose frontal systems are still developing [[20], [21], [22], [23], [24], [25]]. Alternatively, our complementary analysis modeling suicidality as a predictor of insomnia, adjusting for temperature and all covariates, also yielded a significant association, underscoring that directionality cannot be established from these cross-sectional data alone. The temporal sequence of temperature exposure, sleep disruption, and suicidality therefore requires investigation with greater resolution, such as through ecological momentary assessment studies or experimentally in a controlled laboratory environment in which nighttime or daytime temperature is artificially elevated and subsequent sleep and mood effects are observed [42].

Future research should also consider alternative pathway models. For example, it is possible that insomnia might increase exposure to nighttime heat by extending nocturnal wake time, which could affect suicide risk through other mechanisms; however, this pathway was not measured in the current study. Ambient temperature has been correlated with changes in brain functional connectivity [43], changes that have been implicated in increased impulsivity and depressive symptoms [44]. There is also evidence that sleep disruption can influence physical activity levels [45], and decreased physical activity is associated with depressive states [46]. Of note, higher ambient temperatures also have been directly associated with decreases in exercise [47]. It is important to recognize that suicidality clearly arises from multiple interacting and overlapping factors that may not be clearly separable. However, if temperature does influence suicide risk at least partly through sleep disturbance, this presents a promising intervention target, as sleep problems are readily observable, measurable, and modifiable.

### 4.1 Limitations

Multiple limitations of this analysis should be considered. First, our sample includes only youth presenting to an ED. A minority of suicidal youth seek mental health services [48], meaning our sample likely misses many youth whose suicidality may be affected by temperature. Second, interpolated temperature at home addresses only roughly approximates experienced ambient temperatures by patients. We cannot know how much time patients spent indoors or at their home addresses. Mean and minimum daily temperatures also only approximate actual daytime and nighttime temperature exposures. Additionally, we lacked data on access to air conditioning, which may modify the temperature-sleep-suicidality pathway. Third, self-reported sleep disturbance, while comparable to actigraphy for identifying insomnia [49], may underestimate true prevalence [50] and cannot differentiate specific sleep parameters (e.g., sleep onset latency vs. wake after sleep onset) that may be differentially affected by temperature.

Fourth, although we analyzed only initial encounters to ensure each participant was represented once in the dataset and to try to avoid potential confounding from treatment effects or other time-varying factors that might influence subsequent visits, future analyses could employ mixed-effects models to account for repeated visits. Our dataset also did not include current and past psychotropic medications and substance use history, which may impact thermoregulation [51,52]. Future analyses utilizing medical chart data would benefit from inclusion of these covariates in assessing temperature effects on mental health. Fifth, these data were collected during the COVID-19 pandemic, which may have changed individuals’ typical exposures to outdoor temperatures, such as decreasing the amount of time children were physically active outside [53]. Other systemic factors affected by the pandemic, including social isolation, disrupted school, and family stress [54], also impacted depression and suicidality and may affect the generalizability of these findings beyond the pandemic period.

Sixth, the mediation analysis relies on several strong assumptions that may not hold in this cross-sectional design, including the assumption of no unmeasured confounding of the insomnia-suicidality relationship. Although we adjusted for a range of sociodemographic, diagnostic, and environmental covariates, unmeasured factors, such as psychotropic medication use, substance use, and chronic stress, may confound the mediating pathway. Additionally, the cross-sectional design precludes establishing temporal precedence between temperature, insomnia, and suicidality. As demonstrated by our complementary reverse directionality analysis, in which suicidality significantly predicted insomnia after adjusting for temperature and covariates, the direction of this relationship cannot be determined from these data alone and requires prospective investigation. Seventh, suicidality was treated as a single binary outcome encompassing suicidal ideation, suicide attempts, and clinician-assessed suicide risk. Collapsing these heterogeneous presentations into a single variable may obscure differential associations between temperature, insomnia, and specific forms of suicidality, and should be examined in future analyses with sufficient sample sizes to support disaggregated outcomes. Eighth, the dataset was not adequately powered to conduct season-stratified analyses, and season did not emerge as a significant predictor of suicidality in the fully adjusted models. The predominance of winter presentations in this sample may have limited the ability to detect effects that could be most pronounced during summer months, when ambient temperatures are highest.

## 5 Conclusion

In this cross-sectional study of youth presenting to an emergency department in mental health crisis, we provide the first clinical evidence that insomnia moderates and potentially mediates the relationship between ambient heat and suicidality in youth. Temperature was associated with suicidality only among those with insomnia, and minimum temperature, a proxy for nighttime heat exposure, was associated with suicidality through insomnia, suggesting that sleep disruption may represent a clinically meaningful pathway linking ambient heat to acute psychiatric crisis. These findings contribute to the emerging field of climate psychiatry by identifying insomnia as a potentially observable, measurable, and modifiable point of intervention in the heat-suicidality pathway. Clinically, youth presenting to psychiatric emergency services with insomnia during periods of elevated ambient temperature may warrant heightened assessment of suicide risk. With global temperatures continuing to rise, understanding and intervening on the mechanisms linking heat to suicidality in vulnerable populations, particularly youth, is an urgent public health priority. Future mechanistic studies with prospective designs and objective measures of individual-level temperature exposure, sleep, and suicidality are needed to establish causality and develop targeted interventions.

## Declaration of generative AI and AI-assisted technologies in the writing process

During the preparation of this work the authors used Claude.AI 4.6 Sonnet (Anthropic) to help generate a first draft of the code in R Studio for several analyses, which was reviewed and revised as needed by the authors. It was also used to provide suggestions for grammar and clarity of language for select sentences in all sections of this manuscript. After using this tool, the authors reviewed and edited the content as needed and take full responsibility for the content of the published article.

## Role of the funder

None of the funders listed above played any role in the design and conduct of the study; collection, management, analysis, and interpretation of the data; preparation, review, or approval of the manuscript; and decision to submit the manuscript for publication.

## CRediT authorship contribution statement

**Joshua R. Wortzel:** Writing – review & editing, Writing – original draft, Project administration, Methodology, Investigation, Formal analysis, Conceptualization. **Katherine A. Brown:** Writing – review & editing, Methodology, Investigation, Formal analysis, Data curation. **Francis P. Donnelly:** Writing – review & editing, Supervision, Software, Methodology, Data curation, Conceptualization. **Mary Kathryn Cancilliere:** Writing – review & editing, Methodology, Investigation, Data curation. **Elizabeth Chen:** Writing – review & editing, Supervision, Methodology, Funding acquisition, Data curation, Conceptualization. **Kathleen Donise:** Writing – review & editing, Supervision, Methodology, Funding acquisition, Data curation, Conceptualization. **Nicole R. Nugent:** Writing – review & editing, Supervision, Methodology, Investigation, Conceptualization.

## Declaration of competing interest

The authors declare the following financial interests/personal relationships which may be considered as potential competing interests: Joshua Wortzel reports financial support was provided by National Institute of Mental Health. Katherine A. Brown reports financial support was provided by Substance Abuse and Mental Health Services Administration. Elizabeth Chen reports financial support was provided by Substance Abuse and Mental Health Services Administration. Kathleen Donise reports financial support was provided by Substance Abuse and Mental Health Services Administration. Elizabeth Chen reports financial support was provided by NIH National Institute of General Medical Sciences. If there are other authors, they declare that they have no known competing financial interests or personal relationships that could have appeared to influence the work reported in this paper.

## Footnotes

Supplementary material associated with this article can be found, in the online version, at doi:10.1016/j.joclim.2026.100735.
