# Supplementary material for: The influence of temperature on suicidality in youth: The role of insomnia
Source: J Clim Chang Health. 2026 Sep 21;32:100735. doi: 10.1016/j.joclim.2026.100735 (PMC13629221; doi:10.1016/j.joclim.2026.100735)
Supplement: Supplementary file 1 [file mmc1.docx]

**Supplemental Table 1:**

| **Supplemental Table 1A:** |  |  |  |  |  |
| --- | --- | --- | --- | --- | --- |
| **Sequential Model Building Using 7-day Average Daily Mean Temperature** | **Full Models (per 5°C change)** | | | | |
|  | **Estimate** | **OR (RR)** | **RR 95% CI** | **Z score** | **p-value** |
| Model 1 | 0.091 | 1.10 (1.03) | 1.00-1.05 | 2.26 | 0.024 |
| Model 2 | 0.091 | 1.10 (1.03) | 1.00-1.05 | 2.15 | 0.031 |
| Model 3 | 0.12 | 1.13 (1.04) | 1.01-1.06 | 2.67 | 0.008 |
| **Model 4** | 0.12 | 1.13 (1.04) | 1.01-1.06 | 2.67 | 0.008 |
| **Supplemental Table 1B:** |  |  |  |  |  |
| **Sequential Model Building Using 7-day Average Daily Minimum Temperature** | **Full Models (per 5°C change)** | | | | |
|  | **Estimate** | **OR (RR)** | **RR 95% CI** | **Z score** | **p-value** |
| Model 1 | 0.093 | 1.10 (1.03) | 1.00-1.05 | 2.27 | 0.023 |
| Model 2 | 0.092 | 1.10 (1.03) | 1.00-1.05 | 2.17 | 0.030 |
| Model 3 | 0.12 | 1.13 (1.04) | 1.01-1.06 | 2.70 | 0.007 |
| **Model 4** | 0.12 | 1.13 (1.04) | 1.01-1.06 | 2.70 | 0.007 |

**Legend:** Sequential model building with suicidality as the dependent variable. All models contain insomnia and 7-day average daily mean temperature (A) or 7-day average daily minimum temperature (B) and the interaction of insomnia and temperature as independent variables. Model 1 – no other covariates; Model 2 – Model 1 + sociodemographic covariates included (legal sex, minority, age, and primary payer); Model 3 – Model 2 plus mental health diagnostic covariates (depression, anxiety, PTSD); Model 4 – Model 3 plus environmental covariates (average precipitation the 7 days before hospital presentation and season). Model 4 was used for hypothesis testing (bolded). OR indicates the odds ratio, RR the risk ratio, and 95% CI the 95% confidence interval. The reference group for Model 4 was white males less than 14 years old without insomnia with unspecified insurance in winter.
