# Supplementary material for: The influence of temperature on suicidality in youth: The role of insomnia
Source: J Clim Chang Health. 2026 Sep 21;32:100735. doi: 10.1016/j.joclim.2026.100735 (PMC13629221; doi:10.1016/j.joclim.2026.100735)
Supplement: Supplementary file 2 [file mmc2.docx]

**Supplemental Table 2:**

| **Model 4 Negative Controls** | **Per 5 °C change** | | | | |
| --- | --- | --- | --- | --- | --- |
|  | **Estimate** | **OR (RR)** | **RR 95% CI** | **Z score** | **p-value** |
| 7-day Mean Average Temp *Hypersomnia | -0.024 | 0.98 (0.99) | 0.95-1.04 | -0.33 | 0.74 |
| 7-day Mean Average Ppt*Insomnia | -0.040 | 0.96 (0.99) | 0.92-1.05 | -0.36 | 0.72 |

**Legend:** The first row tests the interaction of insomnia and average weekly precipitation with suicidality as the dependent variable. The second row tests the interaction of hypersomnia and weekly average mean temperature with suicidality as the dependent variable. Both models included sociodemographic, mental health, and environmental covariates, as in Model 4. OR indicates the odds ratio, RR the risk ratio, and 95% CI the 95% confidence interval. The reference group for Model 4 was white males less than 14 years old without insomnia with unspecified insurance in winter.
