# Supplementary material for: The influence of temperature on suicidality in youth: The role of insomnia
Source: J Clim Chang Health. 2026 Sep 21;32:100735. doi: 10.1016/j.joclim.2026.100735 (PMC13629221; doi:10.1016/j.joclim.2026.100735)
Supplement: Supplementary file 3 [file mmc3.docx]

**Supplemental Table 3:**

| **Full Model** | **Full Models (per 5 °C change)** | | | | |
| --- | --- | --- | --- | --- | --- |
|  | **Estimate** | **OR (RR)** | **RR 95% CI** | **Z score** | **p-value** |
| Suicidality (Mean Temp Adjusted) | 0.69 | 1.99 (1.63) | 1.48–1.79 | 9.13 | <0.001 |
| Suicidality (Min Temp Adjusted) | 0.69 | 1.99 (1.63) | 1.48–1.79 | 9.12 | <0.001 |

**Legend:** Row 1 models insomnia as the dependent variable with suicidality and 7-day average daily mean temperature as independent variables, in addition to sociodemographic, diagnostic, and environmental covariates. Row 2 uses the same model structure with 7-day average daily minimum temperature. Only the coefficient for suicidality is reported. P-values are Bonferroni corrected. OR indicates the odds ratio, RR the risk ratio, and 95% CI the 95% confidence intervals for the RR. The RR was calculated using the Zhang-Yu approximation formula using the overall prevalence of insomnia in the sample (22.4%) as the baseline. The reference group was white males less than 14 years old without suicidality with unspecified insurance in winter.
